# Intramural Duodenal Hematoma—A Rare Post-Endoscopy Complication in Pediatric Noonan Syndrome: A Case Report

**DOI:** 10.3390/pediatric18040086

**Published:** 2026-06-27

**Authors:** Mariusz Olczyk, Anna Socha-Banasiak, Natalia Lwow, Bartosz Waszczyk, Elżbieta Czkwianianc

**Affiliations:** 1 Department of Gastroenterology, Allergology and Pediatrics, Polish Mother’s Memorial Hospital Research Institute, 93-338 Lodz, Poland; sochabanasiak@gmail.com (A.S.-B.); natalia.a.lwow@gmail.com (N.L.); bartosz.waszczyk@stud.umed.lodz.pl (B.W.); elcia@friend.pl (E.C.); 2 Department of Paediatrics, Immunology and Nephrology, Polish Mother’s Memorial Hospital Research Institute, Medical University of Lodz, 90-419 Lodz, Poland

**Keywords:** Noonan syndrome, intramural duodenal hematoma, pediatric upper gastrointestinal endoscopy, post-endoscopy complication

## Abstract

Background: Noonan syndrome is a rare genetic disorder from the group of RASopathies, characterized by facial dysmorphism, congenital heart defects, hematologic abnormalities, and growth impairment. Case Presentation: We report the case of an 8-year-old girl with Noonan syndrome admitted for evaluation of abdominal pain and failure to thrive. Hematological evaluation before EGD did not identify contraindications to biopsy, and initial laboratory tests, including coagulation parameters, were normal. Several hours after upper gastrointestinal endoscopy, the patient developed abdominal pain and coffee-ground vomiting. Abdominal ultrasonography revealed an intramural duodenal hematoma (58 × 37 mm), which was confirmed and further characterized by computed tomography as an extensive, long-segment lesion involving the duodenum. Progressive anemia required transfusion of blood products. Conservative management, including nasogastric decompression, parenteral nutrition, and pharmacological treatment, was implemented. Despite the severity and prolonged clinical course, gradual clinical and radiological improvement was achieved, and the patient was discharged in good general condition after one month. Conclusions: Intramural duodenal hematoma is an extremely rare complication of upper gastrointestinal endoscopy with duodenal biopsy. This case highlights the importance of individualized assessment and close monitoring in patients with Noonan syndrome, and indicates that this complication should be considered early when abdominal pain, vomiting, or progressive anemia develops after the procedure, even when hematological evaluation and baseline coagulation parameters are reassuring.

## 1. Introduction

Upper gastrointestinal endoscopy, or esophagogastroduodenoscopy (EGD), is performed in pediatric patients only for well-defined clinical indications. Despite appropriate indications, the majority of examinations reveal no significant abnormalities. In a subset of patients, however, the procedure allows for identification of the underlying cause of reported symptoms or abnormalities in growth and development. There are specific groups of patients in whom endoscopy is associated with an increased risk of complications, including younger patients, those with higher American Society of Anesthesiologists (ASA) classification, female sex, and patients undergoing intravenous sedation [1]. Our case of a child with Noonan syndrome highlights a rare but potentially serious complication: an intramural duodenal hematoma.

## 2. Case Presentation

We present the case of an 8-year-old girl with Noonan syndrome (NS) admitted to the Department of Gastroenterology, Allergology, and Pediatrics for the diagnostic evaluation of low body weight, short stature, and recurrent abdominal pain localized to the epigastric and mid-abdominal region. Physical examination revealed facial dysmorphism characteristic of the underlying genetic syndrome. The patient had been diagnosed with Noonan syndrome at the age of 7 years, and endocrinological causes of growth retardation had previously been excluded. These findings raised suspicion of celiac disease or another cause of malabsorption. EGD with duodenal biopsies was planned to assess the duodenal mucosa histologically and to confirm or exclude celiac disease, as the diagnosis would have had direct therapeutic implications. Given the planned duodenal biopsies in a patient with Noonan syndrome, the expected diagnostic benefit of EGD was carefully weighed against the potential procedure-related risks before proceeding.

Before EGD, hematological consultation had been obtained because of the known association between Noonan syndrome and bleeding disorders. According to the available medical documentation, a comprehensive coagulation workup was performed, and von Willebrand disease, hemophilia A and B, clinically relevant coagulation factor deficiencies, thrombocytopenia, and platelet function abnormalities were excluded. The hematologist did not identify contraindications to diagnostic EGD with duodenal biopsy.

Laboratory results on admission were within acceptable ranges for the planned procedure. Hemoglobin level was 12.5 g/dL, hematocrit 37.5%, red blood cell count 4.66 × 10^6^/µL, and white blood cell count 7.49 × 10^3^/µL. Platelet count and standard coagulation parameters were acceptable for the planned procedure: platelet count 171 × 10^3^/µL, activated partial thromboplastin time 30.9 s, prothrombin time 12.9 s, prothrombin index 85.3%, and international normalized ratio 1.2. Given the underlying condition and the potential presence of congenital heart defects, a cardiology consultation was also performed to assess eligibility for general anesthesia. No contraindications to EGD were identified. Abdominal ultrasound revealed only the presence of an accessory spleen measuring 10 mm.

EGD was performed under general anesthesia and revealed a normal endoscopic appearance. As part of the diagnostic workup for suspected celiac disease, five duodenal mucosal biopsy specimens were obtained for histopathological evaluation according to the Marsh–Oberhuber classification: four from the distal duodenum and one from the duodenal bulb. Biopsies were taken using standard single-use biopsy forceps (Single-Use Biopsy Forceps, 2.3 mm; MICRO-TECH Endoscopy, Ann Arbor, MI, USA). The endoscopy was not technically difficult, and no bleeding or other intraprocedural complications were observed. Immediately after the procedure, the girl reported no complaints and was in good condition. Approximately 4 h after the examination, she began to complain of abdominal pain, followed by several episodes of vomiting, initially coffee-ground in character and subsequently with yellow-green content. Physical examination revealed tenderness on palpation in the epigastric region. Peristalsis remained preserved, and no peritoneal signs were present. Due to the patient’s deteriorating condition, a follow-up abdominal and pelvic X-ray was performed, which showed no evidence of perforation.

However, abdominal ultrasonography (US) revealed a hypoechoic, heterogeneous area measuring 58 × 37 mm, without detectable vascular flow, raising suspicion of a partially liquefied, organizing hematoma. The wall of the duodenum was assessed as slightly edematous (Figure 1).

Twelve hours after EGD, a complete blood count was performed, revealing leukocytosis (16.43 × 10^3^/µL), a decreased red blood cell count (3.82 × 10^6^/µL), decreased hemoglobin level (10.5 g/dL), and hematocrit of 29.7%. Platelet count was also reduced to 135 × 10^3^/µL, coagulation parameters were within the normal range; D-dimer levels were also normal (420 ng/mL). Because of the newly diagnosed intramural duodenal hematoma, progressive anemia, and the known association between Noonan syndrome and occult hemostatic abnormalities, antifibrinolytic therapy with tranexamic acid was started. These interventions were based primarily on evolving clinical findings rather than on abnormal baseline coagulation studies. Intravenous ceftriaxone and proton pump inhibitor therapy were introduced according to local practice in the context of a large duodenal wall hematoma, vomiting, and the potential risk of bowel wall compromise. Antiemetic and analgesic treatment was continued. Oral intake was withheld, and total parenteral nutrition was implemented. Blood group was checked twice, and two units of packed red blood cells and fresh frozen plasma were prepared in case of further anemia progression or the need for urgent intervention. The girl was in moderate general condition, with intermittent non-bloody vomiting and tachycardia of up to 130 beats per minute, while arterial blood pressure remained within the normal range.

Approximately 24 h after the EGD, an increasing abdominal circumference and worsening abdominal pain were noted in the patient. Follow-up complete blood count showed further anemia (hemoglobin level—9.1 g/dL, hematocrit 25.5%), and the platelet count was 130 × 10^3^/µL. Due to the suspicion of intestinal obstruction, an attempt was made to perform an upper gastrointestinal contrast study; however, it was discontinued because of recurrent vomiting. Surgical consultation was obtained; however, surgical treatment was not recommended. A decompression tube was inserted, obtaining green content in the drainage bag.

Approximately 48 h after the EGD, follow-up abdominal ultrasound showed further enlargement of the hematoma to 73 × 42 mm. Edema of the duodenal wall with slight blurring of the surrounding fat tissue was described, as well as the appearance of a large amount of fluid in the peritoneal cavity. In follow-up laboratory tests, hemoglobin concentration was 7.5 g/dL and hematocrit was 23.3%. Biphasic abdominal and pelvic computed tomography (CT) was therefore performed, which revealed an extensive, long-segment intramural hematoma involving the distal D2, D3 segments, and D4 of the duodenum, measuring up to 30 mm in D2/D3 and up to 40 mm in D4, with a total length of approximately 20 cm. A large amount of free fluid was noted, most prominently in the right and left lower abdomen, up to 35 mm in the AP dimension. Due to the heterogeneous density of the hematoma dissecting the intestinal wall and the surrounding intraperitoneal fluid, the assessment of bowel wall continuity was significantly limited, and discontinuity could not be excluded (Figure 2).

A repeat abdominal and pelvic X-ray was performed, excluding perforation. Although standard coagulation parameters were not markedly abnormal, transfusion of 2 units of packed red blood cells, 1 unit of fresh frozen plasma, and 1 unit of platelet concentrate was recommended because of progressive anemia, further enlargement of the hematoma, decreasing platelet count, free intraperitoneal fluid, and the high risk of surgical intervention. Platelet concentrate was administered because of the decreasing platelet count and the possibility of qualitative platelet dysfunction associated with Noonan syndrome. Fresh frozen plasma was included as part of supportive hemostatic management in the context of ongoing bleeding and possible urgent invasive treatment. Metronidazole was added to broaden antimicrobial coverage because bowel wall continuity could not be reliably assessed on CT and complicated intra-abdominal pathology remained a concern. To avoid treatment-related changes in laboratory parameters, only values obtained before transfusion of blood products are summarized in Table 1.

In the following days, the patient required continuous analgesic therapy, and antiemetics were administered periodically. She passed stool, although physical examination still revealed decreased peristalsis. After transfusion of blood products, red blood cell parameters nearly normalized (HGB 11.3 g/dL, HCT 31.8%, PLT 210 × 10^3^/µL). Her general condition gradually improved. The decompression tube was maintained, with gravitational drainage of green content without blood admixture. Regular measurements indicated a reduction in abdominal circumference, and follow-up ultrasonography showed no progression of the lesion. Laboratory tests showed no signs of further anemia. Approximately 10 days later, gradual attempts to return to oral feeding were undertaken, but these were repeatedly unsuccessful. On day 14 after the EGD, an upper gastrointestinal contrast study was performed and showed proper passage of contrast through the gastrointestinal tract. Oral feeding was gradually reintroduced, starting with a liquid diet, followed by a soft and easily digestible diet. The patient periodically experienced abdominal pain and transient vomiting, which prolonged the refeeding process and required the introduction of prokinetic treatment (cisapride). Significant clinical improvement occurred only on day 25 after the procedure. Ultimately, after 35 days of hospitalization, the patient was discharged in good general condition for further outpatient care. Follow-up ultrasonography showed no residual hematoma.

## 3. Discussion

Intramural duodenal hematoma is an accumulation of blood within the layers of the duodenal wall (most commonly between the submucosal and muscular layers), leading to narrowing or complete obstruction of the duodenal lumen. In children, abdominal trauma is considered the most common cause of duodenal hematoma [2,3]. This is due to the fact that the duodenum is retroperitoneal in 80–90% of its course and lacks a mesentery, which limits its mobility and makes it more susceptible, compared to other organs, to compression between the anterior abdominal wall and the spine. Its rich vascular supply constitutes another factor increasing the risk. The literature also describes cases associated with neoplasms, anticoagulant therapy and coagulation disorders, pancreatic diseases, as a complication of endoscopy, as well as idiopathic cases [4,5,6,7]. However, the coincidence of two or more potential causes may increase the risk of this very rare complication.

In NS, which is one of the so-called RASopathies, hemostatic disorders represent a relatively common but underestimated problem. In a review paper, Di Candia F. et al. emphasized that bleeding diathesis may affect up to 65% of patients with NS, and platelet dysfunction is considered the most common cause [8]. However, the pathogenesis is multifactorial, and the literature also describes bleeding cases associated with deficiencies of coagulation factors (VIII, XI, XII, XIII), von Willebrand disease, or thrombocytopenia [9,10,11,12]. Moreover, basic coagulation tests often do not correlate with the actual bleeding risk. Therefore, assessment before invasive procedures in patients with NS should not be limited to platelet count, PT/INR, and aPTT, but may include a careful bleeding history and, when indicated, von Willebrand factor testing, selected coagulation factor assays, and platelet function testing. If clinically relevant platelet dysfunction is documented, prophylactic platelet transfusion should be discussed with a hematologist rather than applied routinely. Advanced tools such as thromboelastography (TEG) or the thrombin generation assay (TGA) may help identify patients with an increased bleeding tendency [13,14,15]. Given these limitations, such assays may be considered as part of an individualized hemostatic assessment in selected patients with Noonan syndrome, particularly before high-risk invasive procedures or in those with a bleeding history or abnormal screening results. However, further studies are needed before routine use of these tests can be recommended in this population. In patients with Noonan syndrome undergoing invasive procedures, careful review of bleeding history and available hemostatic assessment remains important, and preventive strategies should be individualized according to clinical risk [15,16,17]. Importantly, recurrent bleeding may be the presenting symptom of Noonan syndrome and may lead to diagnosis even at a later age [18].

Published experience with intramural duodenal hematoma in patients with Noonan syndrome remains very limited. To the best of our knowledge, we identified six similar reports comprising seven patients over the last 25 years [19,20,21,22,23,24]. Only one of the duodenal hematomas occurred spontaneously [22], while the remainder appeared following EGD. Most patients were managed conservatively; half of the cases required parenteral nutrition, and two required the placement of a nasogastric tube. However, in one case described by Ellison et al., the hematoma was managed via arterial embolization, which proved effective and significantly less invasive than surgery [24]. These data are presented in Table 2.

The initial symptoms of an intramural duodenal hematoma may occur within a few hours of the trauma or endoscopic procedure. The clinical picture is dominated by abdominal pain and recurrent vomiting, often leading to symptoms of partial or total gastrointestinal obstruction. Bleeding into the intestinal wall can result in rapidly progressing anemia, as observed in our patient. US is the first-line examination, allowing for a rapid assessment of the duodenal wall. Due to its safety and availability, it allows for regular monitoring of the lesion’s size and early detection of potential complications. Nevertheless, CT remains the diagnostic gold standard [25]. As our case demonstrated, CT more accurately defines the extent of the hematoma and its effect on the intestinal lumen, and helps differentiate it from other complications, such as perforation.

Plain abdominal radiography has limited diagnostic value, although it may help exclude free intraperitoneal air. Monitoring blood count parameters, especially hemoglobin concentration and hematocrit values, plays a key role in the first 24 h. A rapid drop in these parameters indicates the progression of bleeding, which requires escalation of management. In our case, progressive anemia was a direct indication for the transfusion of blood products.

According to a review by Bova et al., conservative treatment is the method of choice for uncomplicated intramural duodenal hematomas, showing high efficacy in hemodynamically stable patients [26]. It primarily includes withholding oral intake, parenteral nutrition, gastrointestinal decompression via a nasogastric tube, analgesic and antiemetic treatment, and protective pharmacotherapy, most commonly with proton pump inhibitors. In situations of rapid blood loss, blood product transfusion may be required to stabilize the patient and allow conservative treatment to continue. Although conservative treatment remains the standard, cases requiring vascular intervention are described in the literature, particularly when hemodynamic stability is absent in patients with Noonan syndrome [24]. Surgical treatment should be reserved for complicated cases, especially in the event of suspected gastrointestinal perforation, persistent obstruction, circulatory instability, or lack of improvement despite treatment. As shown by Ellison et al., selective arterial embolization can be an effective and minimally invasive alternative to laparotomy in situations of active bleeding into the duodenal wall. In our patient, despite the extent of the hematoma and anemia, complete recovery was achieved with conservative management, supporting this approach in hemodynamically stable patients.

Our case shows that normal coagulation parameters before the procedure do not rule out serious bleeding in a patient with Noonan syndrome. The extensive hematoma and prolonged hospitalization illustrate that even diagnostic EGD with biopsy, although minimally invasive, may have a severe and protracted course in this population. Importantly, bleeding occurred despite a clear indication for endoscopy and reassuring hematological assessment. Therefore, patients with Noonan syndrome require individualized risk assessment before invasive procedures and close post-procedural observation; new abdominal symptoms or a decrease in hemoglobin should prompt early evaluation for intramural duodenal hematoma.

## 4. Conclusions

Intramural duodenal hematoma is an exceptionally rare complication of endoscopy, and only isolated cases have been reported in the literature in patients with Noonan syndrome. This case shows that normal baseline coagulation parameters and reassuring hematological assessment do not fully exclude the risk of clinically significant bleeding after biopsy. In patients with Noonan syndrome, abdominal pain, vomiting, or a fall in hemoglobin after EGD should therefore prompt early evaluation for intramural duodenal hematoma. Extended hemostatic assessment may be useful in selected high-risk patients undergoing invasive procedures, although further studies are needed before it can be recommended routinely.

## Figures and Tables

**Figure 1:**
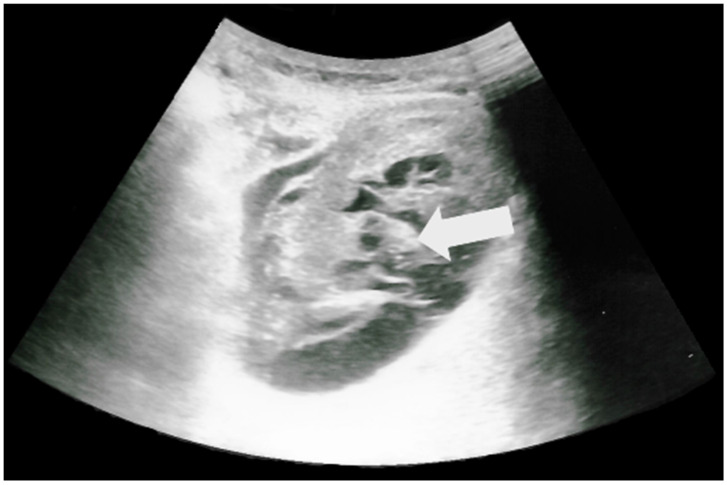
Abdominal ultrasonography showing a heterogeneous hypoechoic lesion measuring 58 × 37 mm, without detectable vascular flow, consistent with a partially liquefied and organizing intramural duodenal hematoma. The arrow indicates the hematoma.

**Figure 2:**
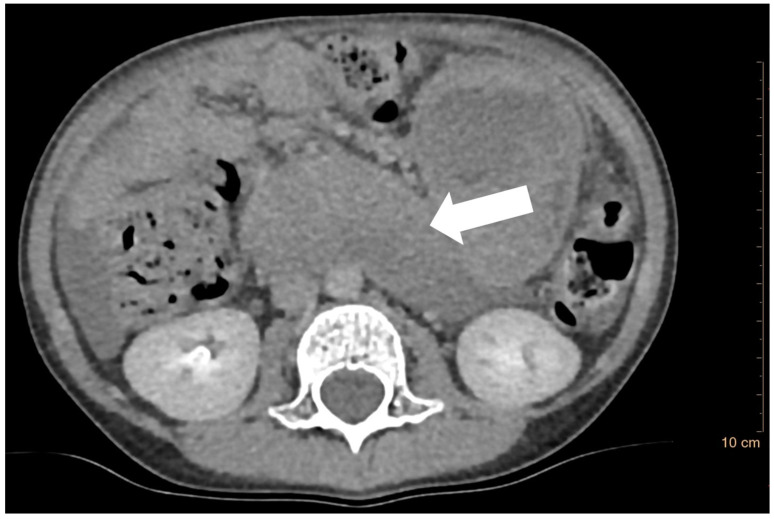
Abdominal computed tomography demonstrating an extensive, long-segment intramural hematoma involving the distal D2, D3, and D4 segments of the duodenum, measuring up to 30–40 mm in width and approximately 20 cm in length, with narrowing of the intestinal lumen and associated free intraperitoneal fluid. The white arrow indicates the lesion.

**Table 1:** Hematological and hemostatic parameters before endoscopy and during the early post-endoscopy course.

Parameter	Before Endoscopy	After Endoscopy—Time from Procedure			Reference Value
		at 12 h	at 24 h	at 48 h	
Hemoglobin [g/dL]	12.5	10.5	9.1	7.5	12–15
Hematocrit [%]	37.5	29.7	25.5	23.3	37–44
PLT ^1^ [10^3^/μL]	171	135	130	113	150–400
PDW ^2^ [fL]	12.2	11.0	10.9	11.7	6.1–11
MPV ^3^ [fL]	10.5	10.1	10.3	10.5	8–10
aPTT ^4^ [s]	30.9	-	28.6	-	23–36.9
PT ^5^ [s]	12.9	-	14.3	-	9.4–12.5
INR ^6^	1.2	-	1.3	-	0.8–1.3
Prothrombin index [%]	85.3	-	76.9	-	80–120
Fibrinogen [g/L]	-	-	2.01	-	2–4.39
Antithrombin III [%]	-	-	101	-	83–128
D-dimer [µg/L]	-	-	420	-	<500

^1^—platelet count; ^2^—platelet distribution width; ^3^—mean platelet volume; ^4^—activated partial thromboplastin time, ^5^—prothrombin time; ^6^—international normalized ratio; “-” means “not measured at this time point”. Post-endoscopy values are shown before transfusion of blood products.

**Table 2:** Summary of published case reports, management, and outcomes.

Year, Country (Author)	Patient	Etiology	Management	Outcome/Additional Findings
2004, Greece (Sgouros et al.) [19]	Adult male (NS ^1^)	Post-biopsy IDH ^2^	Conservative (NG ^4^ tube, PN ^5^, observation)	Recovery after treatment
2011, USA (Vortia et al., abstract) [20]	Two pediatric patients (NS; age/sex not reported)	Post-biopsy IDH	Conservative (observation)	Case 1: hematoma resolved by day 14; Case 2: discharged on day 19; platelet aggregation disorder identified
2012, Italy (Leva et al.) [21]	14-year-old boy (NS)	Post-biopsy IDH	Conservative (NG tube, PN)	Discharge on day 21; factor V, VII, and X deficiency
2018, Japan (Yamazawa et al.) [22]	9-year-old boy (NS)	Spontaneous IDH	Conservative (PN, observation)	Recovery after treatment
2018, Portugal (Reis Sousa et al.) [23]	13-year-old girl (NS + NF1 ^3^)	Post-biopsy IDH	Conservative (observation)	Recovery after treatment; standard coagulation tests normal
2021, Australia (Ellison et al.) [24]	12-year-old boy (NS)	Post-biopsy IDH	Arterial embolization	Surgery avoided; clinical improvement

^1^—Noonan syndrome; ^2^—intramural duodenal hematoma; ^3^—neurofibromatosis type I, ^4^—nasogastric, ^5^—parenteral nutrition.

## Data Availability

The data presented in this study are available on request from the corresponding author.
